# Research Note: Preliminary assessment of the impact of dietary yeast products on egg production and cecal microbial profiles of laying hens

**DOI:** 10.1016/j.psj.2023.102934

**Published:** 2023-07-13

**Authors:** D.R. Korver, S.H. Park, M.K. Costello, E.G. Olson, J.L. Saunders-Blades, S.C. Ricke

**Affiliations:** ⁎ Department of Agricultural, Food, and Nutritional Science, University of Alberta, Edmonton, Canada T6G 2P5; † Department of Food Science and Technology, Oregon State University, Corvallis, OR, 97331, USA; ‡ Department of Animal and Dairy Sciences, Meat Science and Animal Biologics Discovery Program, University of Wisconsin, Madison, WI, 53706, USA

**Keywords:** yeast product, laying hen, cecal microbiota, egg production, eggshell quality

## Abstract

The objective of the current study was to conduct an initial comparison of commercial yeast products in layer hen diets on egg production parameters and the corresponding impact on the cecal microbiota. A short-term feeding study was conducted with 35 laying hens receiving either a control, or 1 of 4 different yeast fermentation products, Immunowall, Hilyses (both from ICC, São Paulo, Brazil), Citristim (ADM, Decatur, IL), and Maxi-Gen Plus (CBS Bio Platforms, Calgary, Canada) with 7 hens per treatment from 40 to 46 wk of age. At the end of the trial, hens were euthanized, the ceca removed and prepared for denatured gradient gel electrophoresis (**DGGE**) microbial compositional analyses. Although initial shell weight and shell thickness were similar among the treatment groups, hens fed Hilyses had lower shell weight and thickness at the end of the experiment. The most predominant DGGE bands with the strongest intensity were identified as *Lactobacillus* species and excised double bands were identified as *Bacillus, Clostridium*, or *Lachnospiraceae.* In this short-term feeding trial, the commercial yeast products tested had little effect on egg production and shell quality, and only moderately impacted the composition of mature layer hen cecal microbiota.

## INTRODUCTION

Yeast fermentation products (by-products and coproducts of various fermentation processes such as the production of ethanol for fuel from cereal grains) are commercially available for inclusion in poultry diets (Roto et al., 2015). Due to the various yeast species and strains used, fermentation conditions, processing methods, and bioactive compound concentrations generated, many different forms of yeast by-products exist. This heterogeneous group of products may elicit different modes of action such as limiting pathogen colonization, immunomodulation and supporting avian gastrointestinal tract (GIT) health (Roto et al., 2015; Ricke, 2018). Numerous studies have examined the impact of various gut health-targeted additives such as prebiotics, probiotics and postbiotics on laying hens, but much less is known about current commercial yeast products on egg production or the GIT microbiota in laying hens (Khan et al., 2020). Therefore, the objective of the current study was to conduct an initial comparison of commercial yeast products with different expected modes of action in laying hen diets on egg production parameters and the corresponding impact on the cecal microbiota. This report is based on a pilot study intended to determine whether various commercially available yeast products impacted egg production by laying hens over a 6-wk period. An initial screening of the impacts of the yeast products on gut microbiota was also conducted.

## MATERIALS AND METHODS

All experimental procedures conformed to the guidelines of the Canadian Council on Animal Care (Canadian Council on Animal Care, 2009) and were approved by the University of Alberta Animal Care and Use Committee: Livestock. A short-term feeding study was carried out at the University of Alberta testing commercial yeast products: Citristim (an inactivated whole cell yeast (*Pichia guilliermondii*) postbiotic; ADM, Decatur, IL) at 0.5 kg/tonne, Maxi-Gen Plus (processed yeast containing nucleotides, β 1,3-glucans and mannans; CBS BioPlatforms Inc., Calgary, Canada) at 1.0 kg/tonne, Hilyses (hydrolyzed product derived from *Saccharomyces cerevisiae*, consisting of peptides, free amino acids, nucleotides, β-glucans and mannanoligosaccharides; ICC, São Paulo, Brazil) at 2.5 kg/tonne, and ImmunoWall (20% mannanoligosaccharide [**MOS**] and 35% β-1-3 glucans from *S. cerevisiae*; ICC, São Paulo, Brazil) at 0.5 kg/tonne*.*

Dietary inclusion level of each yeast product was chosen based on supplier recommendations specific to each product. The primary goal of this study was to assess changes in intestinal microbiota associated with feeding the various products to laying hens and comparing egg performance responses. Single-comb White Leghorn (H & N Nick Chick) laying hens were selected from the larger commercial laying hen population housed at the Poultry Research Centre. Individual hens were selected at random and monitored for a period of 2 wk to ensure that all hens selected were healthy and in production. No selected hen failed to meet these criteria. At the beginning of the experiment, 35 hens were moved to individual laying cages (*n* = 7 per treatment; starting at 40 wk of age) and randomly assigned to one of 5 experimental treatment groups. Hens were fed a standard wheat-based, commercial-type diet (providing 2,750 kcal ME/kg feed, 17.0% CP, 5.0% fat, 4.1% fiber) containing either no yeast product (control) or 1 of the 4 products. Initial (40 wk of age) and final (46 wk of age) body weights, overall feed intake, initial and final egg weights, initial and final shell quality (shell weight and shell thickness) and total egg production from 40 to 46 wk of age were determined for each hen. Egg and shell weights and shell thickness were determined for each egg laid on the first and last days of the experiment, respectively (*n* = 6–7 per treatment). After 6 wk of being on the respective diets, all hens were euthanized, and the ceca were removed using aseptic technique, placed in a sterile Whirl-Pak bag and immediately frozen at −20˚C. The ceca were then shipped to the University of Arkansas on dry ice for cecal microbial profile analysis. Cecal profiles were assessed to indicate changes in microbiota associated with the different commercial yeast products.

### Intestinal Microbiota-PCR-Based Denaturing Gradient Gel Electrophoresis

Genomic DNA from the cecal samples were extracted by a QIAmp Fast DNA Stool mini kit (Qiagen, Valencia, CA) according to the manufacturer's instructions. DNA concentration and purity were measured using a Nanodrop ND-1000 (Thermo Scientific, Wilmington, DE) and subsequently diluted to 25 ng/µL for a polymerase chain reaction (**PCR**) assay prior to denaturing gradient gel electrophoresis (**DGGE**). The PCR assay was conducted following the method described previously (Hanning and Ricke, 2011). A total of 30 µL of PCR reaction volume contained 1 µL of DNA template (25 ng), 333 nM of each forward (CGC CCG CCG CGC GCG GCG GGC GGG GCG GGG GCA CGG GGG GCCTAC GGG AGG CAG CAG) and reverse (ATT ACC GCG GCT GCT GG) primers (Muyzer et al., 1993), 15 µL of Jump Start Ready Mix (Sigma-Aldrich, St. Louis, MO) and 12 µL of DNase-RNase free water. The PCR conditions included a predenaturation step at 95°C for 2 min, followed by 17 cycles of denaturation at 94°C for 1 min, annealing at 67°C for 45 s decreasing by 0.5°C per cycle to a final touchdown temperature of 59°C, and annealing at 72°C for 2 min. The touchdown cycle was then followed by 12 cycles of denaturation at 94°C for 1 min, annealing at 58°C for 45 s with a final extension step of 72°C for 7 min. The PCR products were confirmed on 1.5% agarose by gel electrophoresis and visualized on a transilluminator (Bio-Rad).

The DGGE analyses were conducted as previously described with some modification (Hanning and Ricke, 2011). The polyacrylamide gel used on DGGE included an 8% acrylamide solution and gradient concentration of denaturant. Modification includes adjustment of the denaturant concentration from 60% to 55% and a reduction of electrophoresis time from 17 to 16 h. When the gel was ready, the staining step with SYBR green and the destaining step with distilled water were completed, followed by sequence analysis of the excised bands from the DGGE gel.

Recovery of DNA from the DGGE gel and sequencing were followed as previously described (Hanning and Ricke, 2011). Briefly, targeted bands were excised, disrupted, and resuspended in 300 µL of TE buffer. Samples were vortexed and incubated for 15 min at 65°C. After incubation, samples were filtered by centrifugation in Spin-X tubes (VWR, Radnor, PA). Ethanol precipitation containing 133 µL of 7.5M ammonium acetate, 60 µg of glycogen and 800 µL of 100% ethanol followed centrifugation. Precipitates were vortexed and placed at −70°C for 3 h. The DNA pellets were obtained via centrifugation of the precipitates for 15 min. Pellets were washed with 70% cold ethanol and resuspended in 833 nM of reverse primer. Samples were then submitted to the DNA Resource Center, University of Arkansas, Fayetteville, for sequencing. A phylogenetic tree was then made using Gel Compar II software (Applied Maths, Austin, TX).

### Statistical Analysis

The individually housed hen was the experimental unit for the production parameters. Data were analyzed as a one-way analysis of variance using Proc MIXED of SAS 9.2 (SAS Institute, Cary, NC), with dietary treatment as the main effect. Means were separated using Tukey's test; differences were considered significant at *P* < 0.05.

## RESULTS AND DISCUSSION

### Hen Performance and Egg Characteristics

Initial and final hen body weights, change in body weight, feed intake during the experiment, egg production, and egg weights were not affected by dietary treatment (Table 1). The lack of a treatment effect suggests that, at least in the short term, there were no negative effects of any of the products on hen productivity. The hens were producing as expected over the duration of the experiment (89.6%–94.8% production for the various experimental treatment groups vs. 93.0% for the Control group), and so there may have been little opportunity for the yeast products to increase egg production beyond these levels. Although initial shell weight and shell thickness were similar among the treatment groups, hens fed Hilyses had lower shell weight and thickness at the end of the experiment (*P* = 0.004 and 0.026, respectively). Given the short duration of the trial, the small number of hens in the experiment, and the small numbers of eggs sampled, this could be an experimental artifact and may not reflect an actual decrease in shell quality.

**Table 1:** Performance and shell characteristics of hens fed experimental diets from 40 to 46 wk of age.

Age	Control^1^	Immunowall^2^	Hilyses^3^	Citristim^4^	Maxi-Gen Plus^5^	SEM^6^	*P*-value
	—————————————————————Body weight—————————————————————						
40 wk	1,842.9	1,804.0	1,691.7	1,843.6	1,786.1	69.66	0.537
46 wk	1,874.0	1,830.1	1,701.7	1,859.6	1,797.0	75.77	0.523
	————————————————————Change in body weight (%)^7^————————————————						
	1.41	1.44	0.60	1.07	0.60	0.56	0.980
	—————————————————— Feed Intake (g/bird/d)————————————————————						
	110.7	114.9	108.5	114.0	108.3	4.01	0.680
	————————————————————Egg production (%)————————————————————						
	93.0	92.7	94.8	93.7	89.6	1.91	0.396
	———————————————Number of eggs (40–46 wk of age) ———————————————————						
	38.1	38.0	38.9	38.4	36.7	0.78	0.396
	——————————————————————Egg weight (g)—————————————————————						
40 wk	62.18	64.18	60.48	64.80	62.91	1.65	0.368
46 wk	64.20	63.30	59.83	63.84	63.34	1.93	0.520
	———————————————————Egg shell weight (g)——————————————————————						
40 wk	6.00	6.13	5.72	6.01	5.97	0.14	0.341
46 wk	6.20^a^	6.04^a^	5.18^b^	5.94^a^	6.04^a^	0.18	0.004
	———————————————————Eggshell thickness (mm)————————————————————						
40 wk	0.379	0.380	0.376	0.373	0.373	0.007	0.942
46 wk	0.370^a^	0.365^a^	0.342^b^	0.359^ab^	0.368^a^	0.006	0.026

a,b Means within a row with different superscripts are significantly different.

1 Nutritionally complete diet containing no yeast product.

2 ImmunoWall (40% mannanoligosaccharide and 17% β-1-3 glucans from *S. cerevisiae*; ICC, São Paulo, Brazil) added at 0.5 kg/tonne of complete feed.

3 Hilyses (derived from fermentation of specific strains of *Saccharomyces cerevisiae*, consisting of peptides, free amino acids, nucleotides, β-glucans and mannanoligosaccharides; ICC, São Paulo, Brazil) added at 2.5 kg/tonne of complete feed.

4 Citristim (an inactivated whole cell yeast (*Pichia guilliermondii*) postbiotic; ADM, Decatur, IL) at 0.5 kg/tonne of complete feed.

5 Maxi-Gen Plus (processed yeast containing nucleotides, β 1,3-glucans and mannans; CBS BioPlatforms Inc., Calgary, Canada) added at 1.0 kg/tonne of complete feed.

6 Standard Error of the Mean.

7 Change in individual hen body weight from 40 to 46 wk of age.

### DGGE

**Figure 1:**
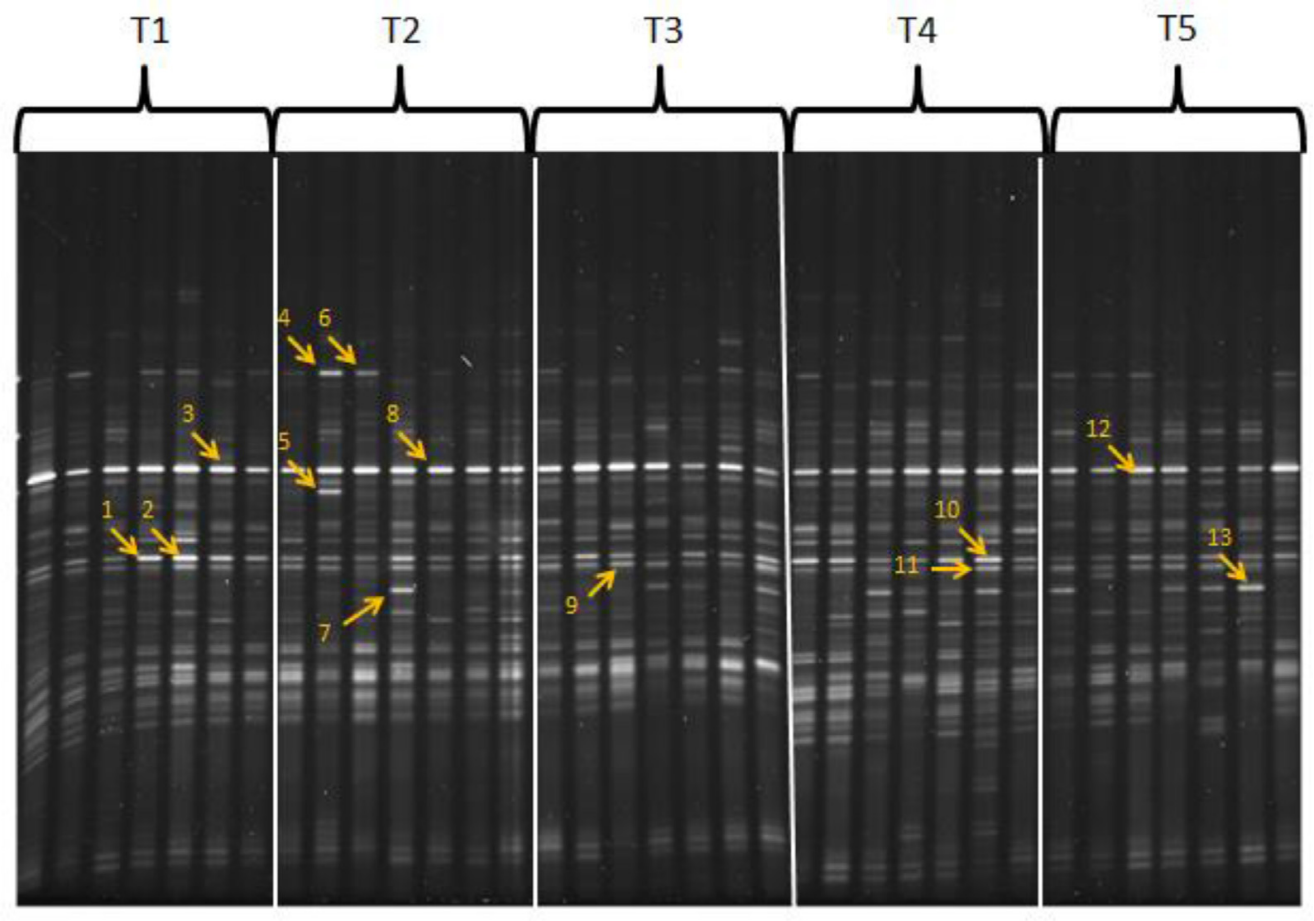
DGGE banding pattern. Seven individual bird sample per treatment; T1 (control), T2 (Immunowall; 40% mannanoligosaccharide and 17% β-1-3 glucans from *S. cerevisiae*; ICC, São Paulo, Brazil; added at 0.5 kg/tonne of complete feed), T3 (Hilyses; derived from fermentation of specific strains of *Saccharomyces cerevisiae*, consisting of peptides, free amino acids, nucleotides, β-glucans and mannanoligosaccharides; ICC; added at 2.5 kg/tonne of complete feed), T4 (Citristim (an inactivated whole cell yeast (*Pichia guilliermondii*) postbiotic; ADM, Decatur, IL) at 0.5 kg/tonne of complete feed) and T5 (Maxi-Gen Plus; processed yeast containing nucleotides, β 1,3-glucans and mannans; CBS BioPlatforms Inc., Calgary, AB, Canada; added at 1.0 kg/tonne of complete feed). Common and specific bands labeled with number were excised for sequencing analysis.

Figure 1 shows DGGE banding patterns from cecal contents of 7 birds per treatment. There was no exclusive band to any specific treatment. When the DGGE banding patterns were compared phylogenetically (data not shown) the patterns generated by all groups exhibited relatedness of 80% with some treatment groups exhibiting even greater relatedness to each other. For instance, hens fed the Citristim and Maxi-Gen Plus products produced DGGE banding patterns that resembled each other more than any of the other groups. Also, the Control and Hilyses groups exhibited distinct phylogenetic clusters; cecal microbiota from hens fed Immunowall were generally shared with the Control and Hilyses groups. These results indicate that while each of the yeast products generated detectable alterations in laying hen cecal microbial profiles, the majority of cecal microbiota were equally impacted by the different yeast-based products relative to the control. This could indicate that the mature layer hen cecal microbiota are fairly well diversified and therefore relatively resilient to dietary modulation in a healthy population of laying hens. This may also help to explain the overall lack of effects on production traits in these high-performing birds. However, it would be interesting to determine if the presence of yeast products accelerated development of the cecal microbial populations as layer hens mature and if this would impact production performance in older laying hens as shell quality decreases with age. For example, earlier DGGE work on broilers fed a yeast fermentate product observed a potential acceleration in development of a stable cecal microbial profile as the birds matured over a 42 d grow-out period (Park et al., 2017).

**Table:** 

Band number	Identified taxonomy
1	*Bacillus*
2, 7, 10, 13	Firmicutes
3, 4, 5, 6, 8, 12	*Lactobacillus*
9, 11	*Clostridium, Lachnospiraceae*

When the distinct bands indicated were excised, several sequenced bands were identified as belonging to the Firmicutes phylum (Figure 1). The most predominant bands with the strongest intensity in the mid region of the gel were identified as *Lactobacillus* species and excised double bands were identified as *Bacillus, Clostridium* or *Lachnospiraceae* (Figure 1). Lee et al. (2016) also recovered several identified *Lactobacillus* spp*.* and *Lachnospiraceae* from sequenced DGGE of ceca from broilers fed a yeast-based prebiotic. Multiple species of *Lactobacillus* are known to colonize the intestinal tract of poultry and also serve as probiotic candidates (Adhikari and Kwon, 2017). Based on the current study it appears that the yeast products tested only moderately altered the composition of mature layer hen cecal microbiota, with minimal subsequent effects on hen performance. In spite of only moderate changes in microbial composition, these changes could still have a considerable impact on cecal fermentation products such as short chain fatty acids and in turn, limit pathogen establishment in the GIT (Ricke et al., 2013). Future studies with yeast fermentation products and other gut health-targeted feed additives for layer hens will require a combination of in-depth microbiome characterization, as well as analysis of fermentative end products and metabolomics to delineate GIT microbial phylogenetic and/or fermentation responses over time as the hens mature.
